# Type of information source and healthcare avoidance: insights from two population-based studies during the COVID-19 pandemic

**DOI:** 10.1186/s12889-025-25927-8

**Published:** 2025-12-20

**Authors:** Marije J. Splinter, Jasmin M. de Groot, Evelien I.T. de Schepper, Pauline W. Jansen, Janine F. Felix, Silvan Licher

**Affiliations:** 1 Department of Epidemiology, Erasmus MC – University Medical Center Rotterdam, Rotterdam, The Netherlands; 2 Generation R Study Group, Erasmus MC – University Medical Center Rotterdam, Rotterdam, The Netherlands; 3 Department of Pediatrics, Erasmus MC – University Medical Center Rotterdam, Rotterdam, The Netherlands; 4 Department of General Practice, Erasmus MC – University Medical Center Rotterdam, Rotterdam, The Netherlands; 5 Department of Child and Adolescent Psychiatry/Psychology, Erasmus MC - University Medical Center Rotterdam, Rotterdam, the Netherlands; 6 Department of Psychology, Education and Child Studies, Erasmus School of Social and Behavioural Sciences, Erasmus University, Rotterdam, the Netherlands

**Keywords:** COVID-19, Healthcare utilisation, Social media, Cohort studies

## Abstract

**Background:**

The COVID-19 pandemic generated major disruptions in primary and specialist care, and there were global trends of reduced healthcare-seeking behaviour. Previous studies showed that use of information sources is linked to psychological well-being and compliance with public health measures. It is unclear if the type of information source is also associated with healthcare avoidance.

**Methods:**

Between April and October 2020, we sent a questionnaire to participants of two population-based cohort studies including middle-aged and older adults from Rotterdam, the Netherlands: the Rotterdam Study (*N* = 8,732, response rate 71.5%) and the Generation R Study (*N* = 4,904, response rate 22.7%). We defined healthcare avoidance as not seeking care despite experiencing symptoms that participants would otherwise interpret as requiring medical attention. We pooled the data from both studies and used logistic regression analyses to examine the adjusted association between type and number of information sources and healthcare avoidance. Additionally, we investigated if symptoms of depression and anxiety modified and/or mediated this association.

**Results:**

Among 6,702 respondents (mean age 67.0 years, 61.8% women), 96.0% used traditional media, 15.5% used social media, and 1,197 (17.9%) avoided healthcare. Compared to non-avoiders, avoiders more often reported poor/fair self-appreciated health (28.9% versus 9.7%), and symptoms of depression (31.9% versus 11.6%) or anxiety (29.2% versus 11.1%). Odds of healthcare avoidance were lower for those who consulted traditional media (adjusted OR (aOR): 0.72, 95% CI: 0.53–1.00) and higher among individuals using social media (aOR: 1.21, 95% CI: 1.02–1.44), compared to those who did not use these sources. Additional adjustment for self-appreciated health and anxiety/depressive symptoms attenuated the latter association. Stratified analyses showed a stronger association between social media use and healthcare avoidance among individuals with anxiety symptoms (aOR: 1.34, 95% CI: 0.94–1.89), yet not statistically significant. Additionally, in a fully adjusted model, part of the association between social media and healthcare avoidance was mediated by anxiety symptoms, although the effect estimate of the mediated effect was small (*p*-value 0.14, natural indirect effect OR: 1.03, 95% CI: 1.01–1.05).

**Conclusions:**

Social media use was associated with higher odds of healthcare avoidance as compared to not using social media, whereas use of traditional media was related to lower avoidance. We found suggestive evidence for a role of anxiety symptoms in these associations, which should be validated by future studies. These findings emphasise the importance of disseminating accessible, objective information across a variety of media platforms, where needed tailored to vulnerable populations, to support informed decision-making during and beyond health crises.

**Supplementary Information:**

The online version contains supplementary material available at 10.1186/s12889-025-25927-8.

## Introduction

 During the COVID-19 pandemic, an abundance of pandemic-related information was disseminated through traditional media outlets (such as radio, television and newspapers), social media and public health institutions [1, 2]. This *infodemic* can be considered a two-sided coin: on the one hand, the widespread availability of information can support forming accurate risk perceptions and making adaptive health decisions [3–5]. On the other hand, continuous exposure to large volumes of information, particularly when conflicting, unverified, or alarming, may induce stress [3, 6–9]. Previous studies have shown that excessive use of social media during the COVID-19 pandemic was related to increased anxiety and depressive symptoms, and vaccine hesitancy [7, 10]. This can result in overestimation of threats, information avoidance, or even reduced compliance with public health measures [3, 5–7]. While these findings suggest that information exposure can influence health behaviours, including healthcare avoidance, it remains unclear whether the use of specific types of information sources is also associated with healthcare avoidance.

Avoidance or delay of medical care can have adverse health consequences. The suspension and downscaling of non-COVID-19-related care led to fewer referrals, diagnoses, and treatments for cancer, cerebrovascular events, and cardiovascular diseases [11–15]. Furthermore, in a previous study, we found that individuals who avoided healthcare during the COVID-19 pandemic for symptoms that would have otherwise made them visit their general practitioner or a medical specialist were at increased risk of all-cause mortality [16]. These individuals were characterised by poorer mental and self-appreciated health [16, 17]. Given that exposure to inaccurate or excessive information can lead to anxiety or depressive symptoms, both of which are associated with healthcare avoidance [17], it is important to determine whether the specific type of information source also contributes to this behaviour. Understanding this association could inform targeted strategies for public communication, particularly to vulnerable individuals, to support access to healthcare during and beyond pandemics.

In this study, we conducted an integrative data analysis of two population-based cohort studies to determine whether the use of specific types of information sources is associated with healthcare avoidance during the COVID-19 pandemic among community-dwelling middle-aged and older adults.

## Methods

### Study population

This population-based study was embedded within two ongoing cohort studies in the city of Rotterdam, the Netherlands: the Rotterdam Study and the Generation R Study. These studies set up nearly identical sub-studies during the COVID-19 pandemic across different age groups, allowing for a wider age range overall when combined.

 The Rotterdam Study is a prospective cohort study aimed at investigating the aetiology and natural history of chronic diseases in mid- and late-life [18]. The Rotterdam Study was initiated in 1990 and consisted of 7,983 residents of the district Ommoord in Rotterdam, the Netherlands, who were 55 years and older (“RS-I”). The cohort was expanded in 2000 (“RS-II”; *N* = 3,011; minimum age 55 years), 2006 (“RS-III”; *N* = 3,932; minimum age 45 years), and 2016 (“RS-IV”; *N* = 3,005; minimum age 40 years). Since 1990, the total study population comprised 17,931 participants, all of whom were extensively examined at study entry and subsequent follow-up every three to six years [18].

The Generation R Study (GenR) is a multi-ethnic, prospective cohort study, designed to identify early environmental and genetic causes and causal pathways leading to normal and abnormal growth, development, and health from fetal life onwards. All pregnant women who had an expected delivery date between April 2002 and January 2006 and living in Rotterdam, the Netherlands, and their partners, were eligible for participation. In total, 9,778 mothers (with or without partner) were enrolled in the study and were invited to complete examinations and questionnaires during pregnancy, and attend follow-up examinations approximately every 4 years after giving birth.

This study is reported according to the Strengthening the Reporting of Observational Studies in Epidemiology (STROBE) guideline.

### COVID-19 questionnaires

#### The Rotterdam Study

On April 8th, 2020, during the first wave of the COVID-19 pandemic in the Netherlands, we identified all participants who were still alive and actively taking part in the Rotterdam Study (*N* = 9,008). Of those, we included participants who were not hospitalized or living in a nursing home in the current study (*N* = 8,732). On April 20th, 2020, we sent these participants a dedicated questionnaire that addressed various aspects of the COVID-19 pandemic, such as COVID-19-related symptoms, socioeconomic factors, mental and physical health, and healthcare utilisation (response rate: 71.5%). A detailed description of the methods of this questionnaire was published elsewhere [19].

#### GenR

During the COVID-19 pandemic, GenR was amidst its 17-year-follow-up measurements. Among a total of 9,778 mothers (with or without partner) who initially participated, 7,394 were still participating in GenR at the 17-year-follow-up. Of these, 4,840 parental units (mother/caretaker and/or partner/father) gave permission to be contacted for additional questionnaires. In October 2020, during the second wave of the COVID-19 pandemic, we invited 3,129 mothers/caretakers and 1774 fathers/partners with a known e-mail address to fill in a digital questionnaire. 1,116 participants responded by the end of December 2020 (response rate: 22.7%). The questionnaire consisted of a total of 333 items, and addressed similar topics as the Rotterdam Study questionnaire, including COVID-19-related symptoms, socio-economic factors, mental and physical health, medication use and healthcare utilisation of the family. The questionnaire was sent digitally using LimeSurvey [20]. We provided an overview of the questionnaire in the Supplementary Material.

### Assessment of information sources

In both studies, we asked participants to indicate whether they obtained information or advice about the COVID-19 pandemic from traditional media outlets (such as television, newspaper, or radio), healthcare institutions (such as the Dutch National Institute for Public Health and the Environment (RIVM) or the World Health Organization), social media (such as Facebook, Twitter, or Instagram), family and friends, or from another source. The Rotterdam Study questionnaire specifically inquired about the previous 14 days, while GenR participants reported on the period from March 2020 to the time of filling in the questionnaire. Participants were able to select multiple answers and specify additional information sources that were not listed by using a free-text comment.

### Assessment of healthcare avoidance

In the Rotterdam Study, we asked participants whether they refrained from seeking medical attention from a general practitioner or medical specialist in the weeks prior to filling out the questionnaire due to the COVID-19 pandemic, despite experiencing symptoms. Those who answered “yes” were considered healthcare avoiders, while “no” respondents formed the reference group. Additionally, we verified healthcare utilisation of a subsample of participants who indicated that they avoided healthcare by checking the medical records of the GP and medical specialists by hand. Detailed information about and results of this verification process are available in a previous study from our group [17]. In the GenR questionnaire, we asked whether participants experienced any symptoms since March 2020 for which they would normally consult a doctor and whether they sought medical attention for these symptoms, with three possible answers: (1) yes, but I did not seek medical care; (2) yes, and I sought medical care, or (3) no, I did not experience such symptoms. Those who gave the first answer were considered healthcare avoiders, while those who answered the other two options formed the reference group.

### Assessment of all-cause mortality and COVID-19 vaccination status

In GenR, information about mortality is not continuously collected and numbers are generally low due to the younger age of the study population. Therefore, we focused on the Rotterdam Study for the analyses of all-cause mortality. The mortality status was available until July 5th, 2024, for all participants of the Rotterdam Study who filled in the question about healthcare avoidance and indicated that they used at least one information source. The date of death was obtained by notification by the municipal administration; general practitioner; nursing home (in case participants moved to a nursing home after filling out the questionnaire); or by the family of the deceased. These follow-up data were complete because of continuous linkage with the Personal Records Database, a national population register maintained by municipalities that contains personal data of people who live in the Netherlands, including date of death. Living status was determined by the most recent date of a research visit, home interview, or MRI scan for the Rotterdam Study; or the last date of checking the medical records from general practitioners by follow-up research assistants, whichever came first. Moreover, through direct linkage with the RIVM we obtained information on COVID-19 vaccination status for Rotterdam Study participants who gave permission for sharing these data. This included the date of vaccination, the type of vaccine received, and the number of booster doses administered, if applicable.

### Assessment of covariates

In addition to age and sex, we considered several variables that we previously identified to be associated with healthcare avoidance as potential confounders [17]: self-reported history of non-communicable diseases (yes if participant had been diagnosed with at least one chronic illness; no), self-appreciated health (excellent; very good; good; fair; poor), educational attainment (primary education; low/intermediate general or lower vocational; intermediate vocational or higher general; higher vocational or university), and occupational status (working; on sick leave; unemployed; retired; other). Additionally, to take into account the difference in timing of the questionnaire distribution and other potential (unmeasured) differences, we also adjusted for cohort in the analyses [21]. We also included symptoms of depression and anxiety, yet, these were measured using different validated scales as part of the study-specific COVID-19 questionnaires: the Rotterdam Study used the weighted score from 10 out of 20 questions on the Centre for Epidemiological Studies Depression (CESD) scale, with a maximum score of 29, while GenR used the weighted (mean) score from 6 items of the Depression domain of the Brief Symptom Inventory-18 (BSI-18), with a mean item score ranging between 0 and 4 [22, 23]. Similarly, the Rotterdam Study assessed symptoms of anxiety using the weighted score from 7 out of 14 questions on the Hospital Anxiety and Depression Scale (HADS), with a maximum score of 20, whereas GenR used the weighted score from 6 items of the Anxiety domain of the BSI-18, with a maximum score of 4 [23, 24]. We harmonised these variables by dichotomising the weighted scores into ‘at risk’ and ‘not at risk’ based on corresponding cut-off scores. In the Rotterdam Study, a weighted score of ≥ 10 signified symptoms of depression and a weighted score of ≥ 7 symptoms of anxiety. In GenR, a weighted score of ≥ 1.17 for men and ≥ 0.83 for women indicated a risk of psychological distress [23].

### Statistical analyses

We performed an integrated data analysis by pooling the data from the Rotterdam Study and GenR [25]. This approach enhanced statistical power and age range of the sample. We reported characteristics of the study population that were measured on a categorical level as frequencies and corresponding percentage of the total number of observations. Only age was measured on a continuous scale, and is presented as median and interquartile range.

We subsequently determined associations between information source use and healthcare avoidance using binary logistic regression analysis, expressed as odds ratios (OR) with 95% confidence intervals, considering both the type and total number of information sources. We compared participants who reported using a specific information source with those who did not use that source, but did use one or more other sources. The total number of sources was categorised as 1, 2 or ≥ 3 information sources to assess whether greater information exposure was related to healthcare-seeking behaviour, given that information overload is associated with stress, anxiety, and depressive symptoms, and subsequent health-related behaviours [3, 6–9]. We constructed the models in three different steps. First, we adjusted for age, sex, and study in Model 1. In Model 2, we additionally adjusted for occupational status, educational attainment and history of any non-communicable disease. Finally, in Model 3, we added self-appreciated health, symptoms of depression, and symptoms of anxiety. As a sensitivity analysis, we also ran these analyses for each cohort separately.

Next, we investigated effect modification to assess whether the strength of the association between type and number of information sources and healthcare avoidance varied according to the presence of anxiety or depressive symptoms. We repeated Models 1, 2 and 3, stratified by either symptoms of anxiety or depression. Additionally, we included interaction terms between each type and number of information sources and symptoms of anxiety or depression to examine their joint effect on healthcare avoidance. Effect modification and interaction are related yet distinct concepts: the former refers to whether the association between type of information source and healthcare avoidance varies across strata of anxiety or depressive symptoms, whereas interaction refers to the joint effect of information source use and anxiety or depressive symptoms on healthcare avoidance [26, 27]. Therefore, we examined both, regardless of the statistical significance of the interaction terms.

Furthermore, since previous studies reported higher levels of psychological distress as a result of using online information sources during the COVID-19 pandemic [3, 7], we investigated whether symptoms of anxiety or depression mediated the relationship between the type and number of information sources and healthcare avoidance. Specifically, we aimed to assess whether these symptoms were on the causal pathway between information source use and healthcare avoidance, rather than considering them as potential confounders that influence both the determinant and the outcome.

We employed causal mediation analysis using the CMAverse package, which is based on the counterfactual framework [28]. This method enables a distinction between direct and indirect effects by following a number of steps using a regression-based approach [29, 30]. First, we calculated the *natural direct effect*, which is the association between information source use and healthcare avoidance that is independent of symptoms of anxiety or depression. Then, we assessed the *natural indirect effect*, representing the part of the association that is mediated by these symptoms. Additionally, the *total effect* was derived by multiplying the natural direct and indirect effects. Finally, we quantified the *proportion of the total effect* that was mediated by symptoms of anxiety and depression by dividing the indirect effect by the total effect. We adjusted for potential confounders of the exposure-outcome, exposure-mediator, and mediator-outcome associations by incrementally adjusting for the covariates mentioned above. We calculated the 95% CI of the odds ratios using bootstrapping with 1,000 replications.

Within the Rotterdam Study, we also examined healthcare avoidance as a mediator in the association between information source use and all-cause mortality, given that previous findings from our group showed an increased risk of all-cause mortality among individuals who avoided healthcare [16].

Missing values in covariates (all less than 3%) were imputed using the missForest package, a non-parametric imputation method based on random forests that can handle both continuous and categorical variables [31]. All analyses were conducted using SPSS version 28.0.1.0 and R version 4.2.2 [32].

## Results

### Characteristics of the study population

Out of a total of 7,357 respondents, 6,702 participants (91.1%) answered the question about healthcare avoidance and reported using at least one information source during the COVID-19 pandemic. Among those, 1,197 (17.9%) individuals avoided healthcare despite experiencing symptoms (Table 1). Compared to the reference group (*N* = 5,505, 82.1%), those who avoided healthcare were more often women (67.1% versus 60.7%), retired (63.7% versus 49.8%), more often had a history of any non-communicable disease (76.9% versus 59.3%), more often considered their health to be poor to fair (28.9% versus 9.7%), more often reported symptoms of depression (31.9% versus 11.6%) and symptoms of anxiety (29.2% versus 11.1%), and had lower COVID-19 vaccination coverage (71.6% versus 79.8%). Supplementary Table 1 provides an overview of cohort-specific characteristics. Of note, 83.6% of GenR participants (*N* = 907) were women.

**Table 1:** Characteristics of the study population

	All (*N* = 6,702)	Healthcare avoiders (*N* = 1,197)	Reference group (*N* = 5,505)
Age (median, IQR)	67.0 (21.0)	71.0 (20.0)	66.0 (21.0)
Women	4,144 (61.8)	803 (67.1)	3,341 (60.7)
Cohort study			
Generation R Study	1,085 (16.2)	70 (5.8)	1,015 (18.4)
Rotterdam Study	5,617 (83.8)	1,127 (94.2)	4,490 (81.6)
**Information sources**	All (*N* = 6,702)	Healthcare avoiders (*N* = 1,197)	Reference group (*N* = 5,505)
Traditional media: newspaper, television or radio	6,435 (96.0)	1,142 (95.4)	5,293 (96.1)
Healthcare institutions (including hospitals/general practitioners)	3,115 (46.5)	519 (43.4)	2,596 (47.2)
Social media	1,036 (15.5)	203 (17.0)	833 (15.1)
Family and friends	1,450 (21.6)	277 (23.1)	1,173 (21.3)
Other*	260 (3.9)	57 (4.8)	203 (3.7)
**Number of information sources**			
One	2,653 (39.6)	485 (40.5)	2,168 (39.4)
Two	2,831 (42.2)	483 (40.4)	2,348 (42.7)
Three	910 (13.6)	172 (14.4)	738 (13.4)
Four	289 (4.3)	54 (4.5)	235 (4.3)
Five	19 (0.3)	3 (0.3)	16 (0.3)
**Educational attainment**			
Primary education	524 (7.8)	113 (9.4)	411 (7.5)
Low/intermediate general or lower vocational	1,946 (29.0)	426 (35.6)	1,520 (27.6)
Intermediate vocational or higher general	1,878 (28.0)	361 (30.2)	1,517 (27.6)
Higher vocational or university	2,354 (35.1)	297 (24.8)	2,057 (37.4)
**Occupational status**			
Working	2,546 (38.0)	270 (22.6)	2,276 (41.3)
On sick leave	96 (1.4)	20 (1.7)	76 (1.4)
Unemployed	238 (3.6)	48 (4.0)	190 (3.5)
Retired	3,504 (52.3)	762 (63.7)	2,742 (49.8)
Other	318 (4.7)	97 (8.1)	221 (4.0)
**History of any non-communicable disease**,** yes**	4,185 (62.4)	921 (76.9)	3,264 (59.3)
**Self-appreciated health**			
Poor	69 (1.0)	39 (3.3)	30 (0.5)
Fair	814 (12.1)	306 (25.6)	508 (9.2)
Good	3,743 (55.8)	665 (55.6)	3,078 (55.9)
Very good	1,511 (22.5)	147 (12.3)	1,364 (24.8)
Excellent	565 (8.4)	40 (3.3)	525 (9.5)
Symptoms of depression**	1,023 (15.3)	382 (31.9)	641 (11.6)
Symptoms of anxiety**	959 (14.3)	350 (29.2)	609 (11.1)
**Vital status (Rotterdam Study only)**	All (*N* = 5,617)	Healthcare avoiders (*N* = 1,127)	Reference group (*N* = 4,490)
Alive on 5 July 2024	5,328 (94.9)	1,036 (91.9)	4,292 (95.6)
Died	289 (5.1)	91 (8.1)	198 (4.4)
**COVID-19 vaccination status (Rotterdam Study only)**			
Received at least one vaccination	4,391 (78.2)	807 (71.6)	3,584 (79.8)
No vaccine registered with RIVM	557 (9.9)	157 (13.9)	400 (8.9)

*Abbreviations: N = *number of participants,* SD = *standard deviation,* RIVM = *the Dutch National Institute for Public Health and the Environment

** The Rotterdam Study: Cut-off score above which individuals are considered to be at risk of clinical depression (weighted score ≥ 10) or anxiety (weighted score ≥ 7), ** The Generation R Study: Cut-off score above which individuals are considered to be at psychological distress (weighted score ≥ 1.17 for men and ≥ 0.83 for women)

Non-responders from the Rotterdam Study were comparable to responders in terms of age (68.9 versus 67.1 years) and sex (61.0% versus 62.2% women), but had fewer years of educational attainment (48.9% versus 31% primary education or low/intermediate general or lower vocational education) and less often had a Northwest-European ethnic background (87.0% versus 94.0%) (Supplementary Table 22). Compared to responders within GenR, GenR non-responders were of similar age (49.7 versus 48.7 years), but included fewer women (56.1% versus 83.8%), fewer individuals with a Northwest-European ethnic background (59.8% versus 78.5%), and a higher proportion of individuals who completed primary, low/intermediate, or lower vocational education (12.3% versus 4.5%).

### Type of information sources and healthcare avoidance

Adjusted for age, sex, and study, the odds of healthcare avoidance were higher among those who used social media compared to those who did not use social media (Table 2, Model 1: OR: 1.27, 95% CI: 1.06–1.52). The effect estimate slightly decreased after further adjustment for occupational status, educational attainment, and history of any non-communicable disease (Table 2, Model 2: OR: 1.21, 95% CI: 1.02–1.44), and attenuated when we additionally adjusted for symptoms of depression, symptoms of anxiety, and self-appreciated health (Table 2, Model 3: OR: 1.13, 95% CI: 0.94–1.36).

**Table 2:** Association between type of information source and healthcare avoidance, additionally stratified by symptoms of anxiety or symptoms of depression

			**Traditional media, yes (reference group: no)**			
	**Yes/no healthcare avoidance (N)**		**OR (95% CI)**			
	Traditional media, no	Traditional media, yes	Model 1	Model 2	Model 3	
Overall association	55/212	1,142/5,293	0.73 (0.53 – 1.00)*	0.72 (0.53 – 1.00)*	0.71 (0.51 – 0.99)*	
Stratified association						*p-*value interaction term
Anxiety symptoms, yes	13/21	337/588	0.87 (0.43 – 1.82)	0.92 (0.45 – 1.94)	0.81 (0.39 – 1.74)	Anxiety x traditional media: *p* = 0.61
Anxiety symptoms, no	42/191	805/4,705	0.67 (0.48 – 0.97)*	0.67 (0.48 – 0.97)*	0.69 (0.49 – 1.01)	
Depressive symptoms, yes	16/22	366/619	0.75 (0.38 – 1.49)	0.74 (0.37 – 1.48)	0.75 (0.37 – 1.53)	Depression x traditional media: *p* = 0.95
Depressive symptoms, no	39/190	776/4,674	0.70 (0.49 – 1.02)	0.70 (0.49 – 1.02)	0.71 (0.49 – 1.04)	

			**Healthcare institutions, yes (reference group: no)**			
	**Yes/no healthcare avoidance (N)**		**OR (95% CI)**			
	Healthcare institutions, no	Healthcare institutions, yes	Model 1	Model 2	Model 3	
Overall association	678/2,909	519/2,596	1.05 (0.92 – 1.19)	1.06 (0.93 – 1.21)	1.05 (0.91 – 1.20)	
Stratified association						*p-*value interaction term
Anxiety symptoms, yes	191/310	159/299	0.95 (0.73 – 1.25)	0.96 (0.72 – 1.26)	1.02 (0.77 – 1.37)	Anxiety x healthcare institutions: *p* = 0.94
Anxiety symptoms, no	487/2,599	360/2,297	1.04 (0.89 – 1.21)	1.05 (0.90 – 1.23)	1.05 (0.89 – 1.23)	
Depressive symptoms, yes	211/339	171/302	1.08 (0.82 – 1.40)	1.05 (0.80 – 1.39)	1.11 (0.83 – 1.47)	Depression x healthcare institutions: *p* = 0.51
Depressive symptoms, no	467/2,570	348/2,294	1.02 (0.87 – 1.19)	1.04 (0.89 – 1.22)	1.02 (0.87 – 1.20)	

			**Social media, yes (reference group: no)**			
	**Yes/no healthcare avoidance (N)**		**OR (95% CI)**			
	Social media, no	Social media, yes	Model 1	Model 2	Model 3	
Overall association	994/4,672	203/833	1.27 (1.06 – 1.52)**	1.21 (1.02 – 1.44)*	1.13 (0.94 – 1.36)	
Stratified association						*p-*value interaction term
Anxiety symptoms, yes	268/491	82/118	1.41 (1.01 – 1.97)*	1.35 (0.96 – 1.89)	1.34 (0.94 – 1.89)	Anxiety x social media: *p* = 0.11
Anxiety symptoms, no	726/4,181	121/715	1.09 (0.87 – 1.34)	1.05 (0.84 – 1.30)	1.04 (0.83 – 1.29)	
Depressive symptoms, yes	305/527	77/114	1.32 (0.94 – 1.84)	1.27 (0.90 – 1.80)	1.23 (0.86 – 1.76)	Depression x social media: *p* = 0.36
Depressive symptoms, no	689/4,145	126/719	1.17 (0.94 – 1.44)	1.13 (0.91 – 1.39)	1.09 (0.87 – 1.34)	

			**Family and friends, yes (reference group: no)**			
	**Yes/no healthcare avoidance (N)**		**OR (95% CI)**			
	Family and friends, no	Family and friends, yes	Model 1	Model 2	Model 3	
Overall association	920/4,332	277/1,173	1.04 (0.89 – 1.21)	1.04 (0.89 – 1.21)	0.94 (0.80 – 1.10)	
Stratified association						*p-*value interaction term
Anxiety symptoms, yes	191/310	159/299	0.96 (0.72 – 1.29)	0.94 (0.69 – 1.26)	0.92 (0.67 – 1.25)	Anxiety x family and friends: *p* = 0.75
Anxiety symptoms, no	487/2,599	360/2,297	0.98 (0.81 – 1.17)	0.98 (0.81 – 1.18)	0.94 (0.78 – 1.13)	
Depressive symptoms, yes	211/339	171/302	0.97 (0.72 – 1.29)	0.93 (0.69 – 1.25)	0.88 (0.65 – 1.19)	Depression x family and friends: *p* = 0.73
Depressive symptoms, no	2,570/467	348/2,294	0.98 (0.81 – 1.17)	0.99 (0.82 – 1.19)	0.96 (0.79 – 1.16)	

			**Any combination of 2 information sources (reference group: no)**			
	**Yes/no healthcare avoidance (N)**		**OR (95% CI)**			
	Use of 1 information source	Any combination of 2 information sources	Model 1	Model 2	Model 3	
Overall association	485/2,168	483/2,348	1.02 (0.89 – 1.18)	1.03 (0.89 – 1.19)	0.98 (0.85 – 1.14)	
Stratified association						*p-*value interaction term
Anxiety symptoms, yes	119/204	119/204	0.95 (0.70 – 1.29)	0.93 (0.68 – 1.28)	0.94 (0.68 – 1.29)	Anxiety x 2 sources: *p* = 0.93
Anxiety symptoms, no	366/1,964	366/1,964	0.98 (0.83 – 1.16)	0.99 (0.84 – 1.17	1.00 (0.84 – 1.18)	
Depressive symptoms, yes	139/215	139/215	0.88 (0.65 – 1.18)	0.87 (0.65 – 1.18)	0.88 (0.64 – 1.19)	Depression x 2 sources: *p* = 0.60
Depressive symptoms, no	346/1,953	346/1,953	1.01 (0.85 – 1.19)	1.02 (0.86 – 1.21)	1.02 (0.86 – 1.21)	

			**Any combination of ≥3 information sources (reference group: no)**			
	**Yes/no healthcare avoidance (N)**		**OR (95% CI)**			
	Use of 1 information source	Any combination of ≥3 information sources	Model 1	Model 2	Model 3	
Overall association	485/2,168	229/989	1.21 (1.01 – 1.45)*	1.20 (1.00 – 1.44)*	1.09 (0.90 – 1.31)	
Stratified association						*p-*value interaction term
Anxiety symptoms, yes	142/855	87/134	1.25 (0.87 – 1.80)	1.23 (0.84 – 1.78)	1.24 (0.85 – 1.82)	Anxiety x ≥3 sources: *p* = 0.32
Anxiety symptoms, no	366/1,964	119/204	1.06 (0.85 – 1.31)	1.05 (0.85 – 1.31)	1.02 (0.82 – 1.27)	
Depressive symptoms, yes	139/215	89/132	1.24 (0.86 – 1.77)	1.18 (0.81 – 1.71)	1.16 (0.79 – 1.68)	Depression x ≥3 sources: *p *= 0.40
Depressive symptoms, no	346/1,953	140/857	1.08 (0.87 – 1.35)	1.09 (0.87 – 1.36)	1.04 (0.83 – 1.30)	

Abbreviations: *N* = number of participants, *OR =* odds ratio

Model 1: adjusted for age, sex and study, Model 2: Model 1, additionally adjusted for occupational status, educational attainment, and history of non-communicable diseases, Model 3: Model 2, additionally adjusted for symptoms of depression, symptoms of anxiety, and self-appreciated health. In the stratified analyses, this model is either adjusted for anxiety or depressive symptoms, and self-appreciated health

*<0.05, **<0.01a Interaction terms are adjusted for age, sex, and study

Conversely, the use of traditional media, as compared to not using traditional media, was associated with lower odds of healthcare avoidance across all models (Table 2, Model 3: OR: 0.71, 95% CI: 0.51–0.99). We did not find an association between use of healthcare institutions or family and friends as information sources and healthcare avoidance, as these effect estimates were consistently near the null. The same applies to using two compared to one information source. Those who used three or more compared to one information source had higher odds of healthcare avoidance (Table 2, Model 2: OR: 1.20, 95% CI: 1.00–1.44), but this estimate declined and lost statistical significance after adjustment for all covariates (Table 2, Model 3: OR: 1.09, 95% CI: 0.90–1.31).

We repeated these analyses stratified by cohort, which yielded similar effect estimates in the Rotterdam Study (Supplementary Table 3). Only the association between social media use and healthcare avoidance was stronger compared to the main analyses (Supplementary Table 3, Model 3: OR: 1.21, 95% CI: 1.01–1.47 versus Table 2, Model 3: OR: 1.13, 95% CI: 0.94–1.36). In GenR, we observed an inverse association between social media use and healthcare avoidance (Supplementary Table 3, Model 3: OR: 0.44, 95% CI: 0.18–0.95), and increased odds of avoidance for those who used healthcare institutions as information source (Supplementary Table 3, Model 3: OR: 1.36, 95% CI: 0.79–2.45). These findings should be interpreted with caution, as small subgroup sample sizes in GenR likely limited the precision of the estimates. To illustrate, in GenR, only seven participants who avoided healthcare also reported using social media (Supplementary Table 1).

### Effect modification by symptoms of anxiety and depression

Compared to no use of social media, social media use was associated with higher odds of healthcare avoidance among individuals who experienced symptoms of anxiety (Table 2, Model 1: OR: 1.41, 95% CI: 1.01–1.97), while we did not find an association in those without anxiety symptoms (Table 2, Model 1: OR: 1.09, 95% CI: 0.87–1.34). Further adjustment for occupational status, educational attainment, history of any non-communicable diseas, symptoms of depression, and self-appreciated health did not substantially change effect estimates in those with symptoms of anxiety, besides loss of statistical significance (Table 2, Model 3: OR: 1.34, 95% CI: 0.94–1.89). Among individuals with depressive symptoms, we observed a similar pattern (Table 2, Model 3: OR: 1.23, 95% CI: 0.86–1.76), yet, none of the models were statistically significant. The association was weaker among those without depressive symptoms (Table 2, Model 3: OR: 1.09, 95% CI: 0.87–1.34).

The use of traditional media was associated with lower odds of healthcare avoidance among individuals who did not report symptoms of anxiety (Table 2, Model 3: OR: 0.69, 95% CI: 0.49–1.01), compared to those who did not use this information source. Effect estimates were weaker in those who did experience symptoms of anxiety (Table 2, Model 3: OR: 0.81, 95% CI: 0.39–1.74).

Adjusted for age, sex, and study, we did not find evidence for multiplicative interaction between type and number of information sources and symptoms of anxiety or depression, with none of the interaction terms being statistically significant (Table 2).

### Mediation by symptoms of depression, symptoms of anxiety and healthcare avoidance

In causal mediation analyses, we observed that the odds of healthcare avoidance were higher among social media users (Table 3, total effect, Model 2: OR: 1.20, 95% CI: 1.02–1.42) than in those who did not use social media, adjusted for age, sex, cohort, occupational status, educational attainment, and history of any non-communicable disease. An estimated 45% (*p-*value: 0.04) of this association was mediated by anxiety symptoms (Table 3, natural indirect effect: OR: 1.08, 95% CI: 1.03–1.15). Further adjustment for symptoms of depression and self-appreciated health attenuated the total effect (Table 3, Model 3: OR: 1.13, 95% CI: 0.95–1.30), and the proportion mediated declined to 22%, which was not statistically significant (Table 3, Model 3: *p-*value 0.14; natural indirect effect, OR: 1.03, 95% CI: 1.01–1.05).

**Table 3:** The association between type of information source and healthcare avoidance, mediated by symptoms of anxiety or symptoms of depression (N = 6,702)

		Model 1 OR (95% CI)	Model 2 OR (95% CI)	Model 3 OR (95% CI)
Mediator: symptoms of anxiety				
Traditional media	Proportion mediated (% (*p-value*)	0% (0.650)	0% (0.582)	0% (0.678)
	Total effect	0.74 (0.56–1.01)	0.73 (0.57–1.00)	0.74 (0.57–0.97)*
	Natural direct effect	0.73 (0.56–0.99)*	0.73 (0.56–0.99)*	0.74 (0.57–0.99)*
	Natural indirect effect	1.03 (0.96–1.08)	1.00 (0.96–1.07)	1.00 (0.98–1.03)
Healthcare institutions	Proportion mediated (% (*p-value*)	61% (0.498)	53% (0.432)	15% (0.476)
	Total effect	1.03 (0.92–1.19)	1.06 (0.93–1.19)	1.05 (0.94–1.17)
	Natural direct effect	1.02 (0.90–1.15)	1.03 (0.91–1.16)	1.04 (0.93–1.17)
	Natural indirect effect	1.01 (1.00–1.06)*	1.03 (1.00–1.06)*	1.01 (1.00–1.02)*
Social media	Proportion mediated (% (*p-value*)	41% (0.008)**	45% (0.038)*	22% (0.140)
	Total effect	1.25 (1.07–1.49)**	1.20 (1.02–1.42)*	1.13 (0.95–1.30)
	Natural direct effect	1.17 (1.00–1.39)	1.13 (0.95–1.33)	1.11 (0.94–1.29)
	Natural indirect effect	1.09 (1.05–1.16)**	1.08 (1.04–1.14)**	1.03 (1.01–1.05)**
Family and friends	Proportion mediated (% (*p-value*)	142% (0.560)	148% (0.686)	0% (0.608)
	Total effect	1.05 (0.91–1.21)	1.04 (0.89–1.20)	0.96 (0.82–1.10)
	Natural direct effect	0.97 (0.85–1.12)	0.97 (0.84–1.12)	0.95 (0.81–1.09)
	Natural indirect effect	1.08 (1.03–1.11)**	1.07 (1.02–1.10)**	1.01 (1.00–1.03)
Any combination of 2 information sources (ref: use of 1 information source)	Proportion mediated (% (*p-value*)	171% (0.710)	105% (0.600)	99% (0.938)
	Total effect	1.02 (0.90–1.18)	1.04 (0.91–1.19)	1.01 (0.88–1.16)
	Natural direct effect	0.99 (0.86–1.13)	1.00 (0.88–1.14)	1.00 (0.88–1.15)
	Natural indirect effect	1.04 (1.01–1.08)**	1.04 (1.01–1.07)**	1.01 (1.00–1.02)
Any combination of ≥ 3 information sources (ref: use of 1 information source)	Proportion mediated (% (*p-value*)	47% (0.032)*	48% (0.040)*	25% (0.234)
	Total effect	1.22 (1.02–1.46)*	1.21 (1.02–1.42)*	1.11 (0.94–1.30)
	Natural direct effect	1.14 (0.96–1.35)	1.12 (0.94–1.32)	1.10 (0.92–1.28)
	Natural indirect effect	1.09 (1.05–1.16)**	1.09 (1.04–1.15)**	1.03 (1.00–1.05)*
Mediator: symptoms of depression				
Traditional media	Proportion mediated (% (*p-value*)	0% (0.774)	0% (0.688)	0% (0.834)
	Total effect	0.74 (0.56–1.02)	0.73 (0.56–1.01)	0.74 (0.57–1.02)
	Natural direct effect	0.73 (0.55–1.01)	0.73 (0.56–0.99)	0.74 (0.57–1.01)
	Natural indirect effect	1.02 (0.95–1.08)	1.00 (0.95–1.08)	1.01 (0.98–1.03)
Healthcare institutions	Proportion mediated (% (*p-value*)	15% (0.606)	7% (0.572)	0% (0.832)
	Total effect	1.04 (0.92–1.20)	1.04 (0.94–1.18)	1.04 (0.92–1.17)
	Natural direct effect	1.03 (0.91–1.18)	1.04 (0.93–1.17)	1.04 (0.92–1.17)
	Natural indirect effect	1.01 (0.98–1.05)	1.00 (0.98–1.04)	1.00 (0.98–1.01)
Social media	Proportion mediated (% (*p-value*)	31% (0.010)	23% (0.034)	0% (0.776)
	Total effect	1.27 (1.06–1.47)**	1.20 (1.02–1.41)*	1.10 (0.95–1.29)
	Natural direct effect	1.20 (1.02–1.39)*	1.16 (0.98–1.35)	1.11 (0.96–1.29)
	Natural indirect effect	1.07 (1.01–1.12)**	1.04 (1.01–1.10)*	0.99 (0.98–1.02)
Family and friends	Proportion mediated (% (*p-value*)	153% (0.624)	171% (0.638)	0% (0.686)
	Total effect	1.04 (0.89–1.21)	1.03 (0.90–1.20)	0.96 (0.83–1.10)
	Natural direct effect	0.98 (0.84–1.12)	0.98 (0.85–1.13)	0.95 (0.82–1.09)
	Natural indirect effect	1.06 (1.03–1.11)**	1.05 (1.02–1.10)**	1.01 (1.00–1.02)
Any combination of 2 information sources (ref: use of 1 information source)	Proportion mediated (% (*p-value*)	154% (0.630)	96% (0.564)	104% (0.928)
	Total effect	1.03 (0.91–1.19)	1.05 (0.92–1.20)	1.00 (0.87–1.15)
	Natural direct effect	0.98 (0.86–1.13)	1.00 (0.88–1.15)	1.00 (0.87–1.14)
	Natural indirect effect	1.04 (1.01–1.08)**	1.05 (1.01–1.07)	1.00 (1.00–1.02)
Any combination of ≥ 3 information sources (ref: use of 1 information source)	Proportion mediated (% (*p-value*)	42% (0.020)*	47% (0.036)*	9% (0.604)
	Total effect	1.23 (1.03–1.48)*	1.24 (1.02–1.44)*	1.10 (0.93–1.28)
	Natural direct effect	1.15 (0.97–1.36)	1.13 (0.95–1.34)	1.10 (0.92–1.28)
	Natural indirect effect	1.09 (1.03–1.16)**	1.10 (1.03–1.13)**	1.01 (0.99–1.03)

Model 1: adjusted for age, sex and study, Model 2: Model 1, additionally adjusted for occupational status, educational attainment, and history of non-communicable diseases, Model 3: Model 2, additionally adjusted for symptoms of depression or symptoms of anxiety, and self-appreciated health

Abbreviations: *N = *number of participants, *OR* = odds ratio

*<0.05, **<0.01

We also observed mediation by symptoms of anxiety in the association between use of healthcare institutions as information source and healthcare avoidance, but the effect estimate of the mediated association was very small (Table 3, natural indirect effect: OR: 1.01, 95% CI: 1.00–1.02). The same applies to those who used three or more compared to one information source (Table 3, natural indirect effect: OR: 1.03, 95% CI: 1.00–1.05).

Symptoms of depression appeared to mediate the association between social media use and healthcare avoidance (Table 3, proportion mediated, Model 2: 23%, *p*-value: 0.03), but the proportion mediated declined to 0% after adjustment for all covariates (Table 3, Model 3: p-value: 0.78). In Supplementary Table 4, we present the mediation analysis of healthcare avoidance in the association between information sources and all-cause mortality. We found no evidence for mediation, as effect estimates for the mediated association were consistently near the null (Supplementary Table 4, natural indirect effects).

## Discussion

### Summary

In this integrated data analysis of two population-based cohort studies, we found that social media use was associated with higher odds of healthcare avoidance, whereas use of traditional media was related to lower avoidance. We observed notable differences in effect estimates suggestive of effect modification by anxiety symptoms, although these were not statistically significant. Furthermore, the association between social media use and healthcare avoidance appeared to be mediated by anxiety symptoms, yet, the effect estimate was very small.

### Comparison with previous studies

Several studies have shown that exposure to misinformation and information overload on social media can increase fear of infection, misperceptions of COVID-19, and overestimation of health risks [33–35]. In turn, these responses may lead to information avoidance and lower adherence to public health recommendations [3, 5–7]. Although the observed indirect effect was small, our finding that symptoms of anxiety partially mediated the relationship between social media use and healthcare avoidance is in line with these previous observations. Notably, the positive association between use of social media and healthcare avoidance appeared stronger among individuals who experienced anxiety symptoms than among those who did not report these symptoms. A possible explanation for these findings is the formation of online echo chambers. An echo chamber is a closed information system or network that isolates and reinforces pre-existing beliefs [36, 37]. This selective exposure is partially driven by social media users themselves, as they choose whom to follow, mainly leading them to content that aligns with their personal views. Additionally, social media algorithms create an echo chamber effect by prioritising posts and articles that users are most likely to engage with based on their online behaviour and interaction [37–39]. During the COVID-19 pandemic, such mechanisms may have intensified exposure to fear-inducing, contradictory, or negative (mis)information, particularly among individuals with anxiety symptoms, as they are more likely to seek information to cope with health-related stress [40–44]. Although some individuals with anxiety symptoms may attempt to seek opposing views for reassurance, this strategy may still expose them to conflicting or distressing information, potentially exacerbating confusion, backlash, and other negative emotional responses [3, 39, 45, 46].

### Implications of our findings

Several strategies have been proposed to mitigate the negative effects of online echo chambers, infodemics, and information overload during (health) crises. These strategies include using accurate and plain language in public health campaigns, addressing public requests for information to identify potential misinformation topics, employing trusted spokespersons or organisations to disseminate information, being aware of the population demographics of the target audience, and leveraging a variety of digital technologies and platforms for information dissemination [47, 48]. The latter strategy might be particularly effective, as our findings suggest that the association between use of traditional media as an information source and healthcare avoidance is weaker among individuals experiencing symptoms of anxiety, which may indicate that traditional broadcasting strategies are less effective in reaching this vulnerable subgroup. Instead, these individuals might be reached through other communication channels. For example, in the Netherlands, the ‘Twijfeltelefoon’ (‘Doubt-Telephone’) was initially established to provide reliable information about COVID-19 vaccines, specifically targeted at those with health-related anxiety [49]. It has since expanded to address questions about other health topics, such as measles, skin cancer and sunscreen use, whooping cough, and contraception [49]. Future research should determine whether these channels are effective in targeting vulnerable, isolated populations.

### Strengths and limitations

A major strength of this study was the direct assessment of healthcare avoidance: we specifically focused on the patient perspective by asking participants whether they decided to avoid healthcare despite having symptoms that would have otherwise prompted them to visit a healthcare provider, rather than relying on indirect data such as consultation rates from medical records, which mainly reflect healthcare utilisation instead of avoidance. Furthermore, by pooling data of two large cohort studies, we were able to investigate the association between information sources and healthcare avoidance in a wide age range of community-dwelling adults.

This study also has several limitations which may have introduced bias into our findings and may limit the extent to which causality can be inferred. First, we did not assess the way or frequency of use of the information sources, while the quality of information can vary within one source. Second, the response rate in GenR was considerably lower than in the Rotterdam Study (22.7% versus 71.5%), likely due to differencs in inclusion criteria and distribution methods. In the Rotterdam Study, all non-institutionalised participants received a questionnaire on paper, whereas in GenR, only participants who had consented to receive additional questionnaires were contacted via e-mail. As a result, some participants may not have been reached, for example due to misdirected e-mails or hesitancy to open an unfamiliar digital link. The distribution periods of the questionnaires for the Rotterdam Study and GenR also differed, namely during the first and second waves of COVID-19 in the Netherlands, respectively. Although these periods were comparable in terms of countermeasures, other differences, such as length of exposure to countermeasures and previous familiarity with the topic, were not specifically controlled for [21]. Yet, including study as a covariate in our analyses did not meaningfully alter effect estimates. Third, the majority of participants were middle-aged or older, White, or had more years of educational attainment, indicating limited generalisability to younger populations, ethnic minority groups, and individuals with fewer years of education. Further research is needed to explore information-seeking behaviour among these underrepresented groups. Finally, we could not include information on whether anxiety and depressive symptoms were pre-existing or developed during the COVID-19 pandemic, due to a long interval between previously measured anxiety symptoms and the COVID-19 questionnaire in the Rotterdam Study. We therefore relied on concurrent assessment of such symptoms with healthcare avoidance in the COVID-19 questionnaire. Future studies in longitudinal cohorts with repeated measurements could examine this in more detail. Such studies could also conduct subgroup analyses among individuals who relied on a single type of information source and avoided healthcare, as the sample sizes of these subgroups were too small in the current study.

## Conclusion

In this integrated data analysis of two population-based cohort studies, we found that those who used social media were more likely to avoid healthcare during the COVID-19 pandemic than those who did not use this information source and that use of traditional media was related to lower healthcare avoidance. We observed suggestive evidence of a role for anxiety symptoms in these associations, but future studies are required to validate these findings. Our findings emphasise the need for consistent information dissemination across various platforms, especially social media, during health crises. Such an approach is crucial for effectively reaching populations in vulnerable situations, improving their access to care, and enhancing their overall well-being.

## Supplementary Information

Supplementary Material 1.

## Data Availability

Data from the Rotterdam Study can be obtained upon request. Requests should be directed towards the management team of the Rotterdam Study (datamanagement.ergo@erasmusmc.nl), which has a protocol for approving data requests. Because of restrictions based on privacy regulations and informed consent of the participants, data cannot be made freely available in a public repository.Data from the Generation R Study are available upon reasonable request to the director of the Generation R Study (generationr@erasmusmc.nl), subject to local, national and European rules and regulations.
