# Supplementary material for: Type of information source and healthcare avoidance: insights from two population-based studies during the COVID-19 pandemic
Source: BMC Public Health. 2025 Dec 20;26:641. doi: 10.1186/s12889-025-25927-8 (PMC12911133; doi:10.1186/s12889-025-25927-8)
Supplement: Supplementary file 1 — Supplementary Material 1. [file 12889_2025_25927_MOESM1_ESM.docx]

**Supplementary Material**

**Type of information source and healthcare avoidance: insights from two population-based studies during the COVID-19 pandemic**

Marije J. Splinter^1*^, Jasmin M. de Groot^2,3*^, Evelien I.T. de Schepper^4^, Pauline W. Jansen^2,5,6^, Janine F. Felix^2,3^, Silvan Licher^1,4^

^1^ Department of Epidemiology, Erasmus MC – University Medical Center Rotterdam, Rotterdam, The Netherlands

^2^ Generation R Study Group, Erasmus MC – University Medical Center Rotterdam, Rotterdam, The Netherlands

^3^ Department of Pediatrics, Erasmus MC – University Medical Center Rotterdam, Rotterdam, The Netherlands

^4^ Department of General Practice, Erasmus MC – University Medical Center Rotterdam, Rotterdam, The Netherlands

^5^ Department of Child and Adolescent Psychiatry/Psychology, Erasmus MC - University Medical Center Rotterdam, Rotterdam, the Netherlands.

^6^ Department of Psychology, Education and Child Studies, Erasmus School of Social and Behavioural Sciences, Erasmus University, Rotterdam, the Netherlands

Corresponding author: Silvan Licher, [s.licher@erasmusmc.nl](mailto:s.licher@erasmusmc.nl)

* Marije J. Splinter and Jasmin M. de Groot contributed equally to this manuscript.

**Content**

**Generation R COVID-19 questionnaire**

**Supplementary Table 1:** Characteristics of the study population stratified by cohort

**Supplementary Table 2:** Characteristics of responders versus non-responders to the questionnaire

**Supplementary Table 3:** Association between information sources and healthcare avoidance, stratified by cohort

**Supplementary Table 4:** The association between information sources and all-cause mortality mediated by healthcare avoidance, within the Rotterdam Study

**Generation R COVID-19 questionnaire**

| Question | Possible answers | Levels/Unit |
| --- | --- | --- |
| What is your DOB? | dd-mm-jjjj | Years |
| What is the highest education program you have finished? | (0) Never went to school or never finished elementary school  (0) Finished elementary school  (0) Finished lower tradeschool  (0) Finished general middle school  (0) Finished general high school  (0) Finished secondary tradeschool  (0) Finished an Applied Sciences degree  (0) Finished a Scientific education | 8 levels (No education (1), Primary school (2), Lower tradeschool (3), Middle school (4), High school (5), Trade school (6), Applied sciences (7), Scientific education (8)) |
| What is your current daily activity/occupation? | (0) I am a student  (0) I am working (full-time, part-time, entrepreneur)  (0) I am on sick leave  (0) I am unemployed  (0) I am retired  (0) I am on maternity leave  (0) I am other: [open answer] | 1 = Stay at home partner/volunteer work, 2 = Sick leave, 3 = Working, 4 = Student, 5 = Unemployed, 6 = Sabbatical,  7 = Maternity leave, NA = N.A. |
| What kind of employment contract do you have? | (0) A permanent contract  (0) Temporary contract  (0) Flexible hours contract  (0) Self-employed  (0) Other | (1) A permanent contract  (2) Temporary contract  (3) Flexible hours contract  (4) Self-employed  (5) Other |
| What is your height in cm? | Height in cm | Cm |
| What is your weight in kilograms? If you own a scale, please use it to answer. | Weight in kilograms (cleaned) | Kgs |
| Did you measure or estimate your weight? | (0) Estimated  (0) Weighed | (1) Estimated (2) Weighed |
| How do you perceive your health, in general? | (0) Bad,  (0) Moderate  (0) Good  (0) Very Good  (0) Excellent | 1 = Bad, 2=Moderate, 3=Good, 4=Very Good, 5=Excellent |
| Did you receive a flu shot in 2019 or 2020? | (0) Yes  (0) No  (0) I don’t know | 1 = Yes, 2 = No, 3 = I don’t know |
| Did you ever receive a tuberculosis vaccine? | (0) Yes  (0) No  (0) I don’t know | 1 = Yes, 2 = No, 3 = I don’t know |
| Would you want to get vaccinated against Covid? | (0) Yes  (0) Maybe  (0) No  (0) I don’t know | 1 = Yes, 2 = Maybe, 3 = No, 4 = I don’t know |
| **Do you have one or more of the following Chronic Illnesses? Please choose the appropriate answer for each part** |  |  |
| Diagnosed with Cardiovascular disease? | (0) Yes  (0) No  (0) Maybe | 1 = Yes, 2 = No, 3 = Maybe |
| Diagnosed with CVA/TIA? | (0) Yes  (0) No  (0) Maybe | 1 = Yes, 2 = No, 3 = Maybe |
| Diagnosed with a Lung disorder? | (0) Yes  (0) No  (0) Maybe | 1 = Yes, 2 = No, 3 = Maybe |
| Diagnosed with Hayfever? | (0) Yes  (0) No  (0) Maybe | 1 = Yes, 2 = No, 3 = Maybe |
| Diagnosed with liver or kidney disease? | (0) Yes  (0) No  (0) Maybe | 1 = Yes, 2 = No, 3 = Maybe |
| Diagnosed with Diabetes Type 1? | (0) Yes  (0) No  (0) Maybe | 1 = Yes, 2 = No, 3 = Maybe |
| Diagnosed with Diabetes Type 2? | (0) Yes  (0) No  (0) Maybe | 1 = Yes, 2 = No, 3 = Maybe |
| Diagnosed with a psychological disorder? | (0) Yes  (0) No  (0) Maybe | 1 = Yes, 2 = No, 3 = Maybe |
| Diagnosed with and Auto-immune disorder? | (0) Yes  (0) No  (0) Maybe | 1 = Yes, 2 = No, 3 = Maybe |
| Diagnosed with Cancer? | (0) Yes  (0) No  (0) Maybe | 1 = Yes, 2 = No, 3 = Maybe |
| Diagnosed with a neurological disorder? | (0) Yes  (0) No  (0) Maybe | 1 = Yes, 2 = No, 3 = Maybe |
| Diagnosed with an impaired immune system? | (0) Yes  (0) No  (0) Maybe | 1 = Yes, 2 = No, 3 = Maybe |
| Have you had health complaints since March 2020 for which you did not contact your physician, even though you would have done so in normal circumstances? | (0) I did not have health complaints for which I normally would have contacted a physician  (0) I did have health complaints, and I contacted my physician  (0) I did have health complaints but I did NOT contact my physician. | 1 = Did NOT have complaints, 2 = Had complaints and went to the doctor, 3 = Care avoidance (had symptoms and did not see a physician) |
| What description best fits these health complaints? | (0) Complaints I had in the past but have not had in a while  (0) Worsening of existing complaints  (0) A new physical complaint  (0) A new psychological complaint (0) Complaints that fit Covid symptoms  (0) Other type of complaint (medication or accident related) | 7 level: 1 = Complaint they had in the past that has now resurfaced,  2 = Worsening of existing symptom  3 = New physical complaint  4 = New psychological complaint  5 = Covid-related/similar  6 = Other  NA = N.A. |
| What is the reason you did not contact a physician for this health complaint, even though you normally would have? | (0) Not serious enough  (0) Sought information elsewhere  (0) Self-treatment  (0) Fear of Covid-19  (0) Did not want to burden doctor  (0) Financial reasons  (0) No time  (0) Other [open answers] | 1 = Not serious enough  2 = Sought information elsewhere  3 = Self-treatment  4 = Fear of Covid-19  5 = Did not want to burden doctor  6 = Financial reasons  7 = No time  8 = Other |
| J7_1_cleaned | No complaints [Did someone in your household/family have health complaints for which they did not contact a docter?] (cleaned) | 0 = No, 1 = Yes |
| Has a family member or someone in your household had health complaints since March 2020 for which they/you did not contact your physician, even though you would have done so in normal circumstances? | (0) No one in the household had health complaints for which I normally would have contacted a physician  (0) One or more household members did have health complaints, and they contacted their physician  (0) One or more household members did have health complaints but they did NOT contact their physician. | 1 = Did NOT have complaints, 2 = Had complaints and went to the doctor, 3 = Care avoidance (had symptoms and did not see a physician) |
| Who was this household member? | (0) My partner  (0) One of the children in our household  (0) More than one child in our household  (0) Another household member | 1 = My partner  2 = One of the children in our household  3 = More than one child in our household  4 = Another household member |
| What description best fits these health complaints? [household member health complaint] | (0) Complaints they had in the past but have not had in a while  (0) Worsening of existing complaints  (0) A new physical complaint  (0) A new psychological complaint (0) Complaints that fit Covid symptoms  (0) Other type of complaint (medication or accident related) | 7 level: 1 = Complaint they had in the past that has now resurfaced,  2 = Worsening of existing symptom  3 = New physical complaint  4 = New psychological complaint  5 = Covid-related/similar  6 = Other  NA = N.A. |
| What is the reason they did not contact a physician for this health complaint, even though they/you normally would have? | (0) Not serious enough  (0) Sought information elsewhere  (0) Self-treatment  (0) Fear of Covid-19  (0) Did not want to burden doctor  (0) Financial reasons  (0) No time  (0) Other [open answers] | 1 = Not serious enough  2 = Sought information elsewhere  3 = Self-treatment  4 = Fear of Covid-19  5 = Did not want to burden doctor  6 = Financial reasons  7 = No time  8 = Other |
| **To what extent did you have the following physical or emotional complaints in the past week?** |  |  |
| Nervousness or shaking? | (0) Not at all,  (0) a little bit,  (0) Somewhat,  (0) Often,  (0) Very often | 1 = Not at all,  2 = a little bit,  3 = Somewhat,  4 = Often,  5 = Very often |
| Easily startled or scared? | (0) Not at all,  (0) a little bit,  (0) Somewhat,  (0) Often,  (0) Very often | 1 = Not at all,  2 = a little bit,  3 = Somewhat,  4 = Often,  5 = Very often |
| Feeling lonely? | (0) Not at all,  (0) a little bit,  (0) Somewhat,  (0) Often,  (0) Very often | 1 = Not at all,  2 = a little bit,  3 = Somewhat,  4 = Often,  5 = Very often |
| Feeling down in the dumps? | (0) Not at all,  (0) a little bit,  (0) Somewhat,  (0) Often,  (0) Very often | 1 = Not at all,  2 = a little bit,  3 = Somewhat,  4 = Often,  5 = Very often |
| No interest in anything anymore/Apathetic? | (0) Not at all,  (0) a little bit,  (0) Somewhat,  (0) Often,  (0) Very often | 1 = Not at all,  2 = a little bit,  3 = Somewhat,  4 = Often,  5 = Very often |
| Feeling fearful/anxious? | (0) Not at all,  (0) a little bit,  (0) Somewhat,  (0) Often,  (0) Very often | 1 = Not at all,  2 = a little bit,  3 = Somewhat,  4 = Often,  5 = Very often |
| Feeling desperate/hopeless about the future? | (0) Not at all,  (0) a little bit,  (0) Somewhat,  (0) Often,  (0) Very often | 1 = Not at all,  2 = a little bit,  3 = Somewhat,  4 = Often,  5 = Very often |
| Feeling nervous/tense? | (0) Not at all,  (0) a little bit,  (0) Somewhat,  (0) Often,  (0) Very often | 1 = Not at all,  2 = a little bit,  3 = Somewhat,  4 = Often,  5 = Very often |
| Anxiety or panic attacks? | (0) Not at all,  (0) a little bit,  (0) Somewhat,  (0) Often,  (0) Very often | 1 = Not at all,  2 = a little bit,  3 = Somewhat,  4 = Often,  5 = Very often |
| Restless to the point of not being able to sit still? | (0) Not at all,  (0) a little bit,  (0) Somewhat,  (0) Often,  (0) Very often | 1 = Not at all,  2 = a little bit,  3 = Somewhat,  4 = Often,  5 = Very often |
| Feelings of worthlessness? | (0) Not at all,  (0) a little bit,  (0) Somewhat,  (0) Often,  (0) Very often | 1 = Not at all,  2 = a little bit,  3 = Somewhat,  4 = Often,  5 = Very often |
| On a scale from 1 to 10, how worried were you about the covid pandemic? [since March 2020] | Score of 1 to 10 | Score of 1 to 10 |
| How often have you been worried that you will get sick? [since March 2020] | (0) Never,  (0) Barely  (0) Sometimes  (0) Often  (0) Always or almost always | 1 = Never,  2 = Barely  3 = Sometimes  4 = Often  5 = Always or almost always |
| How often have you been worried that someone you care about will get sick? [since March 2020] | (0) Never,  (0) Barely  (0) Sometimes  (0) Often  (0) Always or almost always | 1 = Never,  2 = Barely  3 = Sometimes  4 = Often  5 = Always or almost always |
| How often have you been worried that you or your family will get into financial trouble? [since March 2020] | (0) Never,  (0) Barely  (0) Sometimes  (0) Often  (0) Always or almost always | 1 = Never,  2 = Barely  3 = Sometimes  4 = Often  5 = Always or almost always |
| How often have you been worried that you will lose your job? [since March 2020] | (0) Never,  (0) Barely  (0) Sometimes  (0) Often  (0) Always or almost always | 1 = Never,  2 = Barely  3 = Sometimes  4 = Often  5 = Always or almost always |
| How often have you been worried about the consequences of closing the schools for your kids [Since March 2020] | (0) Never,  (0) Barely  (0) Sometimes  (0) Often  (0) Always or almost always | 1 = Never,  2 = Barely  3 = Sometimes  4 = Often  5 = Always or almost always |
| On a scale from 1 to 10, how worried were you about the covid pandemic? [in the past 14 days] | (0) Never,  (0) Barely  (0) Sometimes  (0) Often  (0) Always or almost always | 1 = Never,  2 = Barely  3 = Sometimes  4 = Often  5 = Always or almost always |
| How often have you been worried that you will get sick? [in the past 14 days] | (0) Never,  (0) Barely  (0) Sometimes  (0) Often  (0) Always or almost always | 1 = Never,  2 = Barely  3 = Sometimes  4 = Often  5 = Always or almost always |
| How often have you been worried that someone you care about will get sick? [in the past 14 days] | (0) Never,  (0) Barely  (0) Sometimes  (0) Often  (0) Always or almost always | 1 = Never,  2 = Barely  3 = Sometimes  4 = Often  5 = Always or almost always |
| How often have you been worried that you or your family will get into financial trouble? [in the past 14 days] | (0) Never,  (0) Barely  (0) Sometimes  (0) Often  (0) Always or almost always | 1 = Never,  2 = Barely  3 = Sometimes  4 = Often  5 = Always or almost always |
| How often have you been worried that you will lose your job? [since March 2020] | (0) Never,  (0) Barely  (0) Sometimes  (0) Often  (0) Always or almost always | 1 = Never,  2 = Barely  3 = Sometimes  4 = Often  5 = Always or almost always |
| **Which precautions do you take in order to minimize the spread of the Covid-virus?** |  |  |
| Washing hands often | (0) [Not selected]  (0) Yes | 0 = Not selected, 1 = Yes |
| Using disinfecting (alcohol-based) soaps | (0) [Not selected]  (0) Yes | 0 = Not selected, 1 = Yes |
| Social distancing outside my home | (0) [Not selected]  (0) Yes | 0 = Not selected, 1 = Yes |
| Social distancing inside my home | (0) [Not selected]  (0) Yes | 0 = Not selected, 1 = Yes |
| Covering my nose and mouth in public | (0) [Not selected]  (0) Yes | 0 = Not selected, 1 = Yes |
| Avoid public transport | (0) [Not selected]  (0) Yes | 0 = Not selected, 1 = Yes |
| Avoid travel in general | (0) [Not selected]  (0) Yes | 0 = Not selected, 1 = Yes |
| Self-quarantine | (0) [Not selected]  (0) Yes | 0 = Not selected, 1 = Yes |
| Other precautions taken [open answer] | [Open answer] | N.A. |
| Where do you get information and advice about the Covid virus? [more than one answer possible] | (0) from Media (newspaper, TV or radio)  (0) from Health or Governmental organisations  (0) from Social media  (0) from Friends or Family  (0) Other [open answer] | 1 = from Media (newspaper, TV or radio)  2 = from Health or Governmental organisations  3 = from Social media  4 = from Friends or Family  5 = [non-medical] Work  6 = Other [open answer] |
| N3_cleaned | Did someone you care about get a covid infection? Yes/No (cleaned) | 0 = No, 1 = Yes |
| Did someone you care about die from a covid infection? | (0) Yes  (0) No | 0 = No, 1 = Yes |
| What was your average household income per month before Covid? | (0) <999 euros  (0) 1000-1999,  (0) 2000-2999,  (0) 3000-3999,  (0) 4000-4999,  (0) 5000-5999,  (0) >6000 | 1 = <999 euros  2 = 1000-1999,  3 = 2000-2999,  4 = 3000-3999,  5 = 4000-4999,  6 = 5000-5999,  7 = >6000 |
| What was your average household income per month during Covid (March 2020 until now)? | 0) <999 euros  (0) 1000-1999,  (0) 2000-2999,  (0) 3000-3999,  (0) 4000-4999,  (0) 5000-5999,  (0) >6000 | 1 = <999 euros  2 = 1000-1999,  3 = 2000-2999,  4 = 3000-3999,  5 = 4000-4999,  6 = 5000-5999,  7 = >6000 |
| Gender | (0) Man  (0) Woman  (0) Other | 1 = Man  2 = Woman  3 = Other |

| **Supplementary Table 1. Characteristics of the study population stratified by cohort. Values are numbers (valid percentages) unless stated otherwise.** | | | | |
| --- | --- | --- | --- | --- |
|  | **The Rotterdam Study (N=5,617)** | | **Generation R (N=1,085)** | |
|  | Healthcare avoiders | Reference group | Healthcare avoiders | Reference group |
|  | N=1,127 | N=4,490 | N=70 | N=1,015 |
| **Age (mean, SD)** | 71.6 (12.0) | 68.8 (11.3) | 48.4 (5.3) | 49.8 (4.9) |
| **Women** | 758 (66.3) | 2,506 (55.5) | 59 (84.3) | 848 (83.5) |
| **Information sources** |  |  |  |  |
| Media: newspaper, television or radio | 1,077 (94.2) | 4,340 (96.2) | 65 (92.9) | 940 (94.0) |
| Healthcare institutions (including hospitals/general practitioners) | 467 (40.9) | 1,921 (42.6) | 52 (74.3) | 662 (66.2) |
| Social media | 196 (17.1) | 655 (14.5) | 7 (10.0) | 176 (17.6) |
| Family and friends | 261 (22.8) | 950 (21.1) | 16 (22.9) | 221 (22.1) |
| Other* | 53 (4.6) | 161 (3.6) | 4 (5.7) | 42 (4.1) |
| **Number of information sources** |  |  |  |  |
| One | 471 (41.2) | 1,896 (42.0) | 14 (20.0) | 268 (26.4) |
| Two | 443 (38.8) | 1,847 (40.9) | 40 (57.1) | 492 (48.5) |
| Three | 158 (13.8) | 567 (12.6) | 14 (20.0) | 171 (16.8) |
| Four | 52 (4.5) | 164 (3.6) | 2 (2.9) | 69 (6.8) |
| Five | 3 (0.3) | 16 (0.4) | 0 (0.0) | 0 (0.0) |
| **Educational attainment** |  |  |  |  |
| Primary education | 102 (9.1) | 241 (5.4) | 12 (17.1) | 170 (16.8) |
| Low/intermediate general or lower vocational | 421 (37.4) | 1,453 (32.5) | 5 (7.1) | 55 (5.4) |
| Intermediate vocational or higher general | 355 (31.5) | 1,452 (32.4) | 3 (4.3) | 64 (6.3) |
| Higher vocational or university | 249 (22.1) | 1,330 (29.7) | 50 (71.4) | 723 (71.2) |
| **Occupational status** |  |  |  |  |
| Working | 204 (18.9) | 1,376 (31.1) | 61 (87.1) | 884 (87.2) |
| On sick leave | 16 (1.5) | 45 (1.0) | 4 (5.7) | 29 (2.9) |
| Unemployed | 44 (4.1) | 117 (2.6) | 2 (2.9) | 74 (7.3) |
| Retired | 723 (67.0) | 2,683 (60.7) | 0 (0.0) | 8 (0.8) |
| Other | 92 (8.5) | 197 (4.5) | 3 (4.3) | 19 (1.9) |
| **History of any non-communicable disease, yes** | 889 (77.8) | 2,770 (62.5) | 38 (54.3) | 454 (44.8) |
| **Self-appreciated health** |  |  |  |  |
| Poor | 38 (3.3) | 24 (0.5) | 1 (1.4) | 5 (0.5) |
| Fair | 298 (26.7) | 433 (9.7) | 5 (7.1) | 64 (6.3) |
| Good | 620 (55.5) | 2,576 (58.0) | 41 (58.6) | 480 (47.3) |
| Very good | 125 (11.2) | 1,029 (23.2) | 20 (28.6) | 330 (32.5) |
| Excellent | 36 (3.2) | 380 (8.4) | 3 (4.3) | 135 (13.3) |
| **Symptoms of depression**** | 357 (31.2) | 554 (12.5) | 15 (21.4) | 82 (8.1) |
| **Symptoms of anxiety**** | 340 (29.7) | 549 (12.4) | 12 (17.1) | 54 (5.4) |
| *Abbreviations: N = number of participants, SD = standard deviation*  ** The Rotterdam Study: Cut-off score above which individuals are considered to be at risk of clinical depression (weighted score ≥10) or anxiety (weighted score ≥7).  ** The Generation R Study: Cut-off score above which individuals are considered to be at psychological distress (weighted score ≥1.17 for men and ≥0.83 for women). | | | | |

| **Supplementary Table 2. Characteristics of responders (N=7,357) versus non-responders (N=6,279) to the questionnaire. Values are numbers (valid percentages) unless stated otherwise.** | | | | | |
| --- | --- | --- | --- | --- | --- |
|  | | **Responders** | | **Non-responders** | |
|  | | **Rotterdam Study (N= 6,241)** | **Generation R (N = 1,116)** | **Rotterdam Study (N=2,491)** | **Generation R (N = 3,788)** |
| Age, years (mean, SD) | | 67.1 (10.9) | 49.7 (4.9) | 68.9 (14.0) | 48.7 (5.8) |
| Women | | 4,578 (62.2) | 935 (83.8) | 1,519 (61.0) | 2,125 (56.1) |
| Educational attainment, N (%) |  |  | |  | |
| Primary education | | 351 (4.7) | 7 (0.7) | 304 (12.2) | 101 (3.1) |
| Low/intermediate general or lower vocational | | 1,937 (26.3) | 43 (3.8) | 914 (36.7) | 295 (9.2) |
| Intermediate vocational or higher general | | 2,062 (28.0) | 263 (23.5) | 741 (29.7) | 1,043 (32.7) |
| Higher vocational or university | | 2,361 (32.1) | 762 (75.3) | 513 (20.6) | 1,730 (54.3) |
| Northwest-European ethnic background | | 5,709 (94.0) | 871 (78.5) | 2,085 (87.0) | 2,181 (59.8) |
| *Abbreviations: N, number of participants; SD, standard deviation. All percentages presented are valid percentages.* | | | | | |

| **Supplementary Table 3. The association between information sources and healthcare avoidance, stratified by cohort.** | | | |
| --- | --- | --- | --- |
|  | **Model 1** | **Model 2** | **Model 3** |
|  | **OR (95% CI)** | **OR (95% CI)** | **OR (95% CI)** |
| **The Rotterdam Study (N=5,617)** |  |  |  |
| Traditional media: newspaper, television or radio | 0.71 (0.52 – 1.00)* | 0.69 (0.50 – 0.98)* | 0.68 (0.49 – 0.98)* |
| Healthcare institutions | 1.03 (0.90 – 1.18) | 1.04 (0.91 – 1.20) | 1.03 (0.89 – 1.19) |
| Social media | 1.35 (1.13 – 1.62)** | 1.29 (1.07 – 1.54)** | 1.21 (1.01 – 1.47)* |
| Family and friends | 1.03 (0.88 – 1.21) | 1.03 (0.88 – 1.21) | 0.92 (0.78 – 1.10) |
| Any combination of 2 information sources (ref: use of 1 information source) | 1.01 (0.87 – 1.17) | 1.01 (0.87 – 1.17) | 0.97 (0.83 – 1.13) |
| Any combination of ≥3 information sources (ref: use of 1 information source) | 1.24 (1.02 – 1.50) | 1.21 (1.00 – 1.47) | 1.10 (0.90 – 1.35) |
| **The Generation R Study (N=1,085)** |  |  |  |
| Traditional media: newspaper, television or radio | 0.89 (0.38 – 2.63) | 0.97 (0.40 – 2.93) | 0.88 (0.36 – 2.65) |
| Healthcare institutions | 1.43 (0.84 – 2.55) | 1.40 (0.82 – 2.51) | 1.36 (0.79 – 2.45) |
| Social media | 0.48 (0.20 – 1.01) | 0.50 (0.20 – 1.04) | 0.44 (0.18 – 0.95)* |
| Family and friends | 1.01 (0.55 – 1.77) | 0.99 (0.54 – 1.75) | 0.94 (0.50 – 1.67) |
| Any combination of 2 information sources (ref: use of 1 information source) | 1.58 (0.86 – 3.07) | 1.60 (0.87 – 3.13) | 1.54 (0.83 – 3.04) |
| Any combination of ≥3 information sources (ref: use of 1 information source) | 1.24 (0.59 – 2.64) | 1.25 (0.59 – 2.67) | 1.14 (0.53 – 2.45) |
| *Abbreviations: N, number of participants; OR, odds ratio*  *<0.05; **<0.01  Model 1: adjusted for age and sex  Model 2: model 1, additionally adjusted for occupational status, educational attainment and history of any non-communicable disease  Model 3: model 2, additionally adjusted for symptoms of depression, symptoms of anxiety, and self-appreciated health | | | |

| **Supplementary Table 4. The association between information sources and all-cause mortality mediated by healthcare avoidance, within the Rotterdam Study (N=5,617).** | | | |
| --- | --- | --- | --- |
|  | Model 1 | Model 2 | Model 3 |
|  | OR (95% CI) | OR (95% CI) | OR (95% CI) |
| **Traditional media** | | | |
| Proportion mediated (% (*p*-value)) | 18% (0.646) | 9% (0.628) | 7% (0.792) |
| Total effect | 0.87 (0.55 – 1.64) | 0.86 (0.54 – 1.58) | 0.90 (0.58 – 1.52) |
| Natural direct effect | 0.90 (0.57 – 1.73) | 0.87 (0.55 – 1.65) | 0.88 (0.57 – 1.53) |
| Natural indirect effect | 0.97 (0.95 – 1.00) | 0.98 (0.96 – 1.00) | 0.99 (0.97 – 1.01) |
| **Healthcare institutions** | | | |
| Proportion mediated (% (*p*-value)) | 0% (0.964) | 0% (0.994) | 0% (0.844) |
| Total effect | 0.76 (0.61 – 0.92)* | 0.76 (0.62 – 0.92)* | 0.78 (0.65 – 0.94)* |
| Natural direct effect | 0.76 (0.61 – 0.92)* | 0.76 (0.62 – 0.91)* | 0.78 (0.65 – 0.94)* |
| Natural indirect effect | 1.00 (0.99 – 1.01) | 1.00 (0.99 – 1.01) | 1.00 (0.99 – 1.01) |
| **Social media** | | | |
| Proportion mediated (% (*p*-value)) | 0% (0.890) | 1% (0.862) | 4% (0.724) |
| Total effect | 0.87 (0.61 – 1.15) | 0.85 (0.60 – 1.17) | 0.86 (0.61 – 1.13) |
| Natural direct effect | 0.86 (0.60 – 1.13) | 0.84 (0.60 – 1.16) | 0.86 (0.61 – 1.13) |
| Natural indirect effect | 1.01 (0.96 – 1.06) | 1.00 (0.96 – 1.04) | 0.99 (0.96 – 1.02) |
| **Family and friends** | | | |
| Proportion mediated (% (*p*-value)) | 0% (0.978) | 0% (0.980) | 0% (0.844) |
| Total effect | 1.01 (0.81 – 1.24) | 0.99 (0.80 – 1.18) | 0.95 (0.78 – 1.13) |
| Natural direct effect | 1.00 (0.81 – 1.24) | 0.99 (0.80 – 1.18) | 0.95 (0.78 – 1.14) |
| Natural indirect effect | 1.00 (0.99 – 1.01) | 1.00 (0.99 – 1.01) | 1.00 (0.99 – 1.02) |
| **Any combination of 2 information sources (ref: use of 1 information source)** |  |  |  |
| Proportion mediated (% (*p*-value)) | 0% (0.970) | 0% (0.974) | 0% (0.888) |
| Total effect | 0.89 (0.74 – 1.07) | 0.86 (0.72 – 1.04) | 0.85 (0.71 – 1.02) |
| Natural direct effect | 0.88 (0.74 – 1.06) | 0.86 (0.72 – 1.04) | 0.85 (0.81 – 1.02) |
| Natural indirect effect | 1.01 (0.99 – 1.01) | 1.00 (0.99 – 1.01) | 1.00 (0.99 – 1.02) |
| **Any combination of ≥3 information sources (ref: use of 1 information source)** |  |  |  |
| Proportion mediated (% (*p*-value)) | 6% (0.336) | 14% (0.304) | 11% (0.538) |
| Total effect | 0.82 (0.60 – 1.07) | 0.80 (0.58 – 1.05) | 0.78 (0.58 – 1.03) |
| Natural direct effect | 0.80 (0.58 – 1.05) | 0.78 (0.58 – 1.04) | 0.77 (0.57 – 1.02) |
| Natural indirect effect | 0.99 (0.94 – 1.01) | 0.97 (0.94 – 1.01) | 0.97 (0.95 – 1.02) |
| *Abbreviations: N, number of participants; OR, odds ratio.* *<0.05  Model 1: adjusted for age and sex  Model 2: model 1, additionally adjusted for occupational status, educational attainment and history of any non-communicable disease  Model 3: model 2, additionally adjusted for symptoms of depression, symptoms of anxiety, and self-appreciated health | | | |
